# Oral losartan yields subtherapeutic airway exposure compared to nebulized delivery and fails to improve mucociliary clearance in cystic fibrosis

**DOI:** 10.3389/fphar.2026.1807581

**Published:** 2026-05-04

**Authors:** Charles D. Bengtson, Andreas Schmid, Michael D. Kim, John S. Dennis, J. Steven Leeder, Kirby L. Zeman, William D. Bennett, Juan Sabater, Nathalie Baumlin, Matthias Salathe

**Affiliations:** 1 Department of Internal Medicine, Division of Pulmonary Critical Care and Sleep Medicine, University of Kansas Medical Center, Kansas, KS, United States; 2 Department of Pediatrics and Children’s Mercy Research Institute, Children’s Mercy Hospital, Kansas, MO, United States; 3 Department of Medicine, University of North Carolina at Chapel Hill, Chapel Hill, NC, United States; 4 Department of Research, Mount Sinai Medical Center, Miami Beach, FL, United States; 5 Department of Internal Medicine, University of Kansas Medical Center, Kansas, KS, United States

**Keywords:** cystic fibrosis, distinct pharmacokinetics, inflammation, inhaled vs. oral, losartan, mucociliary clearance, TGF-β1

## Abstract

**Background:**

Transforming growth factor-β1 (TGF-β1) contributes to more severe pulmonary disease in people with cystic fibrosis (pwCF). Our preclinical *in vitro* and *in vivo* studies demonstrated that losartan could reverse TGF-β1-mediated mucociliary dysfunction, albeit at doses difficult to achieve by oral administration. This study therefore examined airway losartan concentrations after oral and inhaled dosing to evaluate if oral losartan could be even expected to improve mucociliary clearance.

**Methods:**

We conducted comparative pharmacokinetics studies in sheep assessing airway surface liquid (ASL) concentrations of losartan and its metabolites, EXP3179 and EXP3174, after oral and inhaled administration. Another pharmacokinetic study used oral losartan to compare serum levels in pwCF on and off CFTR modulators and healthy volunteers. Finally, an open-label clinical trial evaluated effects of oral losartan (50 mg twice daily for 14 weeks) on mucociliary and cough clearance (MCC/CC) in pwCF off CFTR modulators as they have a need for novel therapies (NCT03435939).

**Results:**

In sheep, oral losartan (50 mg twice daily for 4 days) did not achieve ASL levels expected to improve CF-associated mucociliary dysfunction based on our previous data. In humans, single dose pharmacokinetics of oral losartan showed differential results in pwCF and healthy volunteers, with pwCF off CFTR modulators achieving the lowest serum levels of losartan and EXP3174 with a complete absence of EXP3179, the anti-inflammatory metabolite of losartan. The open label study included seven participants. Serum concentrations of losartan and EXP3174 at week 14 matched levels observed in pwCF not on modulators in the pharmacokinetic study. As expected, no improvements in whole-lung or peripheral MCC/CC were found in participants with complete datasets. PpFEV1 remained unchanged, but nasal TGF-β1 levels significantly decreased with treatment, although systemic inflammatory markers were unaffected (C-reactive protein, calprotectin, and serum amyloid A).

**Conclusion:**

Reduced drug exposure compared to healthy volunteers reflects altered pharmacokinetics in pwCF. Nevertheless, oral losartan at clinically approved doses does not achieve airway concentrations needed to restore mucociliary function in any individual, and especially not in pwCF off modulators. Future studies should consider inhaled drug delivery to achieve therapeutically meaningful airway exposure to losartan and its metabolites.

## 1 Introduction

Close to 40,000 people with Cystic Fibrosis (pwCF) live in the United States (Foundation, 2025). Historically, lung disease with respiratory failure caused mortality, driven by mucus hyperconcentration, mucociliary dysfunction, chronic infection, and airway inflammation (Cohen-Cymberknoh et al., 2013; Ratjen et al., 2015). CF Transmembrane Conductance Regulator (CFTR) modulator therapy has improved outcomes for eligible pwCF by improving lung function, decreasing sweat chloride, reducing exacerbations, and improving quality of life (Ramsey et al., 2011; Middleton et al., 2019; Keating et al., 2025). However, no similar therapies exist for the ∼10% of pwCF with two CFTR variants not amenable to modulators. Across trials and registries, ∼15–20% of eligible pwCF have minimal percent predicted forced expiratory volume in 1 s (ppFEV1) improvement on elexacaftor/tezacaftor/ivacaftor. Nevertheless, those low FEV1 responses (<5%) can show benefits in lung clearance index (when baseline lung function is high), exacerbations, BMI, infection status, and quality of life (Heijerman et al., 2019; Middleton et al., 2019; Zemanick et al., 2021; Nichols et al., 2022).

Before modulators, chronic inflammation led to permanent structural damage to the airways and a progressive decline in lung function (Roesch et al., 2018). This is still the case for pwCF off modulators. Even with modulator therapy, inflammation is not fully resolved to levels seen in healthy volunteers (Schaupp et al., 2023; Harris et al., 2020; Casey et al., 2023; Schaupp et al., 2023). However, clinical trials assessing anti-inflammatory therapies in pwCF have shown limited or no sustained clinical benefit (Elborn et al., 2021; Konstan et al., 2025; West et al., 2025). Azithromycin, often used in those with chronic *Pseudomonas aeruginosa* infections, is thought to have some anti-inflammatory properties and ibuprofen is effective to slow lung function deterioration in children (Konstan et al., 1995; Konstan, 2008; Konstan et al., 2018). The latter is not used commonly (Konstan et al., 2010). Thus, novel anti-inflammatory medications are still needed to treat all pwCF regardless of modulator therapies.

The inflammatory signature of the CF airway is heterogeneous. If dominated by transforming growth factor-beta1 (TGF-β1), the inflammation is associated with more severe pulmonary disease (Arkwright et al., 2000; Drumm et al., 2005; Harris et al., 2011; Kramer and Clancy, 2018). TGF‐β1 reduces rescue of F508del CFTR by modulators (Snodgrass et al., 2013; Sun et al., 2014) and impairs the activity of additional ion channels important for airway mucus hydration, including the large conductance, calcium-activated potassium (BK) channel (Kim et al., 2020; Kim et al., 2022). Previous studies demonstrated that TGF-β1 impairs mucociliary function in human cystic fibrosis bronchial epithelial cells (CFBE) by reducing BK channel activity by reducing the expression of leucine-rich repeat-containing protein 26 (LRRC26), which is required for BK function in the airway epithelium (Manzanares et al., 2015; Kim et al., 2020). These detrimental effects were reversed with clinically relevant concentrations of the angiotensin II receptor blocker (ARB) losartan (Kim et al., 2020; Kim et al., 2022), albeit concentrations that were difficult to achieve using oral therapy even at the maximum FDA-approved dose. In a previously established CF-like sheep model, using TGF-β1or CFTR_inh_172 to inhibit CFTR plus human neutrophil elastase to induce inflammation (including TGF-β1) reduced tracheal mucus velocity, a surrogate for mucociliary clearance, providing a physiologically relevant large animal model for assessing airway drug exposure pertinent to pwCF (Kim et al., 2020). The CFTR_inh_172 plus human neutrophil elastase effects were reversed by losartan inhalation, achieving calculated airway concentrations above published plasma levels with oral administration (Kim et al., 2020).

Losartan is a prodrug that is metabolized to an aldehyde intermediate (EXP3179), which is further metabolized to a carboxylic acid (EXP3174). EXP3174 has a potent angiotensin II receptor type 1 (ATR1) blocking activity (Sica et al., 2005). EXP3179, on the other hand, exerts anti-inflammatory properties with no ATR1-blocking activity (Kramer et al., 2002). The ability of losartan to restore mucociliary function in CFBE cells *in vitro* and a sheep model of CF-like disease *in vivo* were demonstrated to be independent of angiotensin II receptor blockade and instead attributable to losartan’s anti TGF‐β1–mediated modulation of BK channel function (Kim et al., 2020; Kim et al., 2022).

Given losartan’s safety profile and anti-inflammatory effects in CF models, our study tested its efficacy to improve mucociliary clearance and cough clearance (MCC/CC) in pwCF. Because of concerns of reaching sufficient airway concentrations using the oral route, we explored multiple avenues, including measuring the pharmacokinetics of oral losartan in normal volunteers and pwCF, assessing airway concentrations of losartan and its metabolites in aqueous distribution volume of ovine airways after inhalation of losartan, and evaluating changes in nasal TGF-β1 expression and mucociliary clearance/cough clearance (MCC/CC) in pwCF not taking modulators as this group would benefit the most from this intervention.

## 2 Materials and methods

### 2.1 Study overview

This work consisted of three complementary study components.

- a large animal pharmacokinetic study in a CF-like sheep designed to quantify airway concentrations of losartan and its metabolites following oral versus inhaled delivery;
- a human serum pharmacokinetic study comparing healthy volunteers with pwCF on or off CFTR modulator therapy; and
- an open label exploratory clinical trial evaluating whether oral losartan could plausibly improve mucociliary or cough clearance in pwCF not receiving modulator therapy.

### 2.2 Measurements of airway exposures to losartan and its metabolites in sheep

All animal experiments were conducted in accordance with institutional and national guidelines for the care and use of laboratory animals and were approved by the Mount Sinai Medical Center Institutional Animal Care and Use Committee (IACUC). Study design and animal use adhered to the principles of the 3 R s: Replacement (large animal studies were undertaken only after extensive *in vitro* work in CFBE cells and only because airway dosing and mucus sampling cannot be adequately modeled *in vitro*), Reduction (the number of sheep, n = 3 per condition, was minimized and based on prior studies using the same CF-like sheep model that demonstrated reproducible pharmacokinetic and mucociliary outcomes with comparable group sizes), and Refinement (sheep were studied while conscious during aerosol delivery to avoid anesthetic-related respiratory effects, and all procedures were performed to minimize stress and discomfort). Adult healthy female sheep were used and monitored throughout all procedures as published (Chung et al., 2019; Danahay et al., 2020; Kim et al., 2020).

Different sheep were used for oral and inhaled dosing protocols. These experiments were designed as pharmacokinetic and exposure assessment studies rather than hypothesis testing efficacy experiments. Therefore, formal power calculations were not performed. However, previous experimentation with this model showed that n = 3 was having sufficient power due to low variability.

#### 2.2.1 Airway concentrations of losartan and its metabolites after oral losartan dosing

After collecting tracheal mucus *in vivo* directly from the tracheal surface using suction catheters without dilution techniques, oral losartan (50 mg daily) was provided to individual sheep in the drinking water for 3 days (n = 3). On day 4, tracheal mucus was collected 1, 2, 4, 6, 8, and 24 h after a last dose of oral 50 mg losartan.

At certain post dose time points, only small amounts of airway mucus were available due to the limited airway surface liquid volume and variability of mucus production. To ensure reliable quantification of losartan and its metabolites by UPLC MS/MS in the tracheal mucus samples without artificially stimulating secretion, collected secretions from adjacent time points were pooled: samples from 2 and 4 h as well as 6 and 8 h were combined and designated as 3 h and 7h, respectively.

#### 2.2.2 Airway concentrations of losartan and its metabolites after nebulized losartan dosing

Sheep were conscious and intubated (n = 3). Aerosols were delivered using dosimetry. A respirator delivered aerosols directly during inspiration at a rate of 20 breaths/min and a tidal volume of 500 mL. Losartan (50 mg) was freshly dissolved in 2 mL sterile phosphate buffered saline without additional excipients. Tracheal mucus was collected before and 1, 2, 4, 6, 8, and 24 h after the inhaled dose was delivered. Since some timepoints yielded only small amounts of mucus, we combined samples from 2 and 4 h as well as 6 and 8 h and designated them 3 h and 7 h, respectively.

Prior to experimentation, expected airway concentrations were estimated assuming a unitless deposition fraction of 0.1 and an ovine aqueous airway distribution volume of 20 mL, based on published measurements (Wanner et al., 1988). The assumed deposition fraction of 0.1 was intentionally conservative and accounts for anticipated losses during aerosol generation and delivery. With these parameters, nebulizing 50 mg losartan into sheep airways should result in an immediate concentration of at least ∼550 µM. This estimate was used to contextualize measured airway drug concentrations. Although mass median aerodynamic diameter (MMAD) was not directly measured in this study, the nebulizer configuration, dosimetric delivery, and controlled breathing parameters were selected based on prior ovine aerosol studies using the same equipment that achieved efficient lower-airway deposition in sheep.

Losartan, EXP3174, and EXP3179 concentrations recovered in airway mucus after nebulization were quantified using validated UPLC MS/MS. The detection of both metabolites at high airway concentrations (see results) demonstrates chemical stability of losartan during aerosolization and rapid airway metabolism.

This CF-like sheep model reproduces key airway features relevant to cystic fibrosis, including impaired mucociliary clearance due to mucus hyperconcentration and airway inflammation following CFTR inhibition and application of neutrophil elastase (commonly found in CF airways). It does mimic the chronic infection and biofilm formation but is nevertheless a commonly used testbed for drugs aimed at improving mucociliary clearance.

### 2.3 Human serum pharmacokinetics in healthy volunteers and pwCF on and off modulators

A pharmacokinetic (PK) study was completed in pwCF on and off modulators (4 participants for on and 3 for off modulators) as well as healthy volunteers (3 participants), collecting samples hourly over eight hours after a single 50 mg dose of losartan. CFTR variants are listed in Supplementary Table S1.

### 2.4 Open label trial of oral losartan in pwCF off modulators

#### 2.4.1 Study design

We conducted an open-label clinical trial of treatment with oral losartan (50 mg daily for 1 week followed by 100 mg daily for 13 weeks) in pwCF off modulators. The study protocol was approved by the University of Kansas Medical Center IRB, and written informed consent was obtained from each participant. The study was registered on ClinicalTrials.gov (NCT03435939).

A schematic of the clinical trial design is shown in Supplementary Figure S1. Adults (≥18 years of age) with a known diagnosis of CF and not on modulator therapy were enrolled in the study. Subjects were excluded if they had a ppFEV1 of <40% at screening visit or unstable lung disease (defined by a change in medical regimen during the preceding 2 weeks or a ppFEV1 ≥15% below average of 3 months prior to enrollment). Subjects on ARBs or angiotensin-converting enzyme (ACE) inhibitors, or who showed intolerance to ARBs, were also excluded from the study. Other exclusion criteria included pregnancy, regular use of nonsteroidal anti-inflammatory drugs (NSAIDs) or aliskiren, oral corticosteroid use within 6 weeks, exacerbation requiring treatment within 6 weeks, treatment of mycobacterial infections, significant hypoxemia, untreated arterial hypertension, blood pressure <90 mmHg systolic while standing, known renal artery stenosis, concomitant airway disorders other than CF, and radiation exposure within the past year that would cause them to exceed Federal regulations by participating in the study. A complete list of eligibility criteria can be found in Supplementary Material.

The primary endpoint was change in MCC/CC measured by gamma scintigraphy between baseline and after 14 weeks of losartan. Secondary endpoints included changes in percent predicted forced expiratory volume in 1 s (ppFEV1), CFQ-R scores, levels of inflammatory markers (serum: high sensitive C-reactive protein, serum amyloid A, calprotectin, nasal fluid TGF-β1 and tissue necrosis factor alpha (TNF-α) as well as nasal cell LRRC26 mRNA expression (BK *γ* subunit, required for BK function in non-excitable cells).

#### 2.4.2 MCC/CC measurements

MCC/CC measurements were done in line with prior studies (Bennett et al., 2013). Subjects inhaled an aerosol of sulfur colloid (SC) labeled with ∼40 µCi technetium-99 m (Tc99 m) according to previously published protocols (Donaldson et al., 2006; Bennett et al., 2011; Bennett et al., 2013). The Tc99 m-SC particles in isotonic solution were placed in an adjusted Pari LL nebulizer (PARI, Starnberg, Germany) delivering 8.5 µm particles. A closed delivery system was used to produce a very slow inspiratory airflow (55 mL/s) determined by the compressor flow rate to the nebulizer. Participants performed about six to ten single inhalations monitored with visual feedback using a magneheliec pressure meter targeting between 0.5 and 1 inch of water and then gently exhale targeting about 0.5 inches of water on the same system. The gamma camera was utilized during inhalation to monitor dosing to the lung with the subject positioned in front of the camera. Continuous 2-min images were recorded for 90 min. During the first hour, subjects were encouraged to suppress spontaneous coughing. To assess cough clearance (CC) from 60–90 min, subjects voluntarily coughed 30 times through a peak flow meter, dispersed evenly over the 30 min. Spontaneous cough frequency was also recorded.

Whole lung clearance is dependent on the site of particle deposition (Ilowite et al., 1989; Bennett et al., 2013). To characterize regional deposition in the lung, a transmission scan was obtained to outline the area of the whole lung. Americium fiducial markers were used to allow accurate image alignment. Only the right lung was used for analysis of both regional deposition and MCC/CC because of tracer accumulation in the stomach. Central (C) vs. peripheral (P) deposition was determined by regions of interest (ROI) analysis (Ilowite et al., 1989; Donaldson et al., 2006; Donaldson et al., 2007; Bennett et al., 2010; Bennett et al., 2011; Bennett et al., 2013) and normalized to C/P for the transmission scan. Whole lung ROI of the right lung was used to determine retention over the 90-min clearance period. Average clearance from 0–60 min for MCC and between 60-90 min for CC was calculated by averaging retentions at 10 min intervals.

#### 2.4.3 Cystic fibrosis questionnaire-revised (CFQ-R)

The CFQ-R is a CF-specific health-related quality of life (HRQOL) measure that evaluates 12 domains, including physical functioning, role perception, vitality, emotion, social perception, body image, eating disturbance, treatment burden, health perception, weight, respiratory symptoms, and digestive symptoms (Quittner et al., 2005; Quittner et al., 2012). The CFQ-R was administered to study participants at baseline and after 14 weeks of losartan treatment.

#### 2.4.4 Human nasal fluid collection (leukosorb)

Collection of nasal epithelial lining fluid in human participants was performed using pre-cut strips of absorptive filters (Leukosorb; Pall Corporation, Port Washington, NY, United States) as previously described (Kim et al., 2022).

#### 2.4.5 Nasal cell collection

Nasal cells were collected using sterile cytology brushes (Medical Packaging Corporation, Camarillo, CA, United States) as previously described (Bengtson et al., 2021; Kim et al., 2021; Kim et al., 2022).

#### 2.4.6 Enzyme-linked immunosorbent assay (ELISA)

C-reactive protein (CRP) was measured from plasma samples, and TGF-β1 and TNF-α were measured from nasal epithelial lining fluid samples using the Ella™ Automated ELISA and Simple Plex Cartridges (Bio-Techne, Minneapolis, MN, United States). Calprotectin and serum amyloid A (SAA) were measured with Invitrogen Human Calprotectin L1/S100-A8/A9 Complex ELISA Kit (Cat. No. EH62RB; Thermo Fisher Scientific) and Invitrogen Human SAA ELISA kit (Cat. No. KHA0011; Thermo Fisher Scientific), respectively.

#### 2.4.7 Droplet digital PCR (ddPCR)

RNA was isolated from nasal epithelial cells using the E. Z.N.A.® Total RNA Kit (Omega Bio-tek, Norcross, GA, United States) and cDNA was made using the iScript™ cDNA synthesis kit (Bio-Rad, Hercules, CA, United States). ddPCR was performed using the ddPCR Supermix for Probes (Bio-Rad) and TaqMan primers for *LRRC26* (Hs2385555_g1; Thermo Fisher Scientific, Waltham, MA, United States) and *GAPDH* (4326317 E; Thermo Fisher Scientific). Droplets were generated using a QX100™ Droplet Generator (Bio-Rad) and PCR was performed with a C1000 Thermal Cycler (Bio-Rad). Droplets were read using a QX100™ Droplet Reader (Bio-Rad) and data were analyzed with QuantaSoft™ software (Bio-Rad).

#### 2.4.8 Drug concentration measurements

Concentrations of losartan, losartan carboxylic acid (EXP3174), and losartan carboxaldehyde (EXP3179) from all samples were analyzed by ultra-performance liquid chromatography tandem mass spectrometry (UPLC-MS/MS) with positive ion electrospray ionization using multiple reaction monitoring (MRM). Data acquisition and analysis were performed using MassLynx 4.2 software (Waters).

#### 2.4.9 Statistical analysis

Baseline demographics and clinical characteristics were summarized using descriptive statistics. Mean difference and 95% confidence intervals for continuous variables were estimated using Student’s t-test. MCC/CC measurements were analyzed using two-way ANOVA.

## 3 Results

### 3.1 Pharmacokinetic study of oral and inhaled losartan in sheep

#### 3.1.1 Airway concentrations of losartan and its metabolites after oral losartan dosing (50 mg daily for 4 days)

**FIGURE 1:**
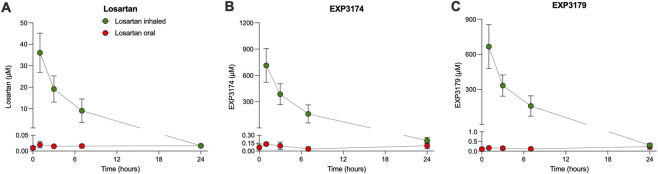
Measurements of airway exposures to losartan and its metabolites after oral and nebulized administration in ewes. Sheep (each n = 3) were exposed to nebulized (50 mg) or oral losartan (4 days, 50 mg each) and airway mucus was collected before and after treatments (for timepoints see methods) **(A)** Measurement of losartan in airway surface mucus (green) showed a concentration peak of 36 μM, below the theoretically calculated minimal 550 µM given a deposition fraction of 0.1 and a sheep airway surface liquid of 2 mL. This means that losartan is rapidly metabolized. Oral losartan (red) did not reach any significant concentration in the airway surface liquid **(B)** The angiotensin receptor blocking metabolite EXP3174 is found at high concentrations, peaking 1 h after the losartan dose at >700 µM. Oral losartan did not result in any significant concentration of EXP3174 in the airway surface liquid **(C)** The anti-inflammatory metabolite EXP3179 is found at high concentrations, peaking 1 h after the losartan dose at >600 µM. Oral losartan did not result in any significant concentration of EXP3179 in the airway surface liquid.

Collected tracheal secretions were analyzed for losartan and its metabolites (Figure 1, red dots) after oral dosing of 50 mg per day for 4 days. As seen in Figure 1, tracheal secretion concentrations of losartan never exceeded 0.04 µM, a concentration far below the minimally effective concentrations of ≥1 µM losartan used for *in vitro* effectiveness in bronchial cells from pwCF (Kim et al., 2020; Kim et al., 2022). In addition, the levels of EXP3174 and 3,179, the latter being the anti-inflammatory metabolite, remained low in the tracheal secretion samples. EXP3179 reached a maximum of 0.4 µM (Figure 1). Thus, these levels remained below the ones likely required for improving mucociliary dysfunction related to inflammation in pwCF as shown in cell culture experiments, including the ones measuring conversion from losartan to EXP3179 (Supplementary Figure S3E in Kim et al., 2020).

#### 3.1.2 Airway concentrations of losartan and its metabolites after nebulized losartan dosing (50 mg, one dose)

Nebulizing losartan (50 mg) into sheep airways resulted in high airway concentrations of losartan and both its metabolites (Figure 1, green dots). As stated above, the expected losartan concentration in the aqueous distribution compartment in the airways was calculated to be at least 550 µM with an assumed deposition fraction of 0.1 and an ovine aqueous compartment of 20 mL, with higher concentrations possible with increasing deposition fraction increased. The data show that losartan was rapidly and locally metabolized into EXP3179 and EXP3174 with high concentrations of these two metabolites persisting for >8 h, consistent with efficient formation and/or preferential airway retention. Concentrations of losartan and its metabolites, including EXP3179, fell below 1 µM only at the 24 h post dosing measurement point. Given our previously published data, such concentrations were associated with improved ASL volume and reduced mucus concentrations *in vitro* and improved mucociliary clearance *in vivo* (Kim et al., 2020; Kim et al., 2022).

### 3.2 Pharmacokinetic study of oral losartan in pwCF and healthy volunteers

**FIGURE 2:**
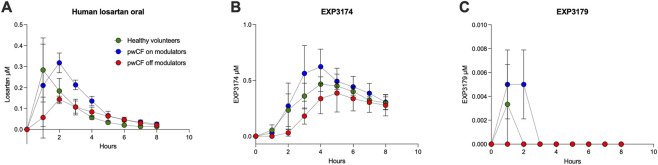
Serum pharmacokinetics of oral losartan in pwCF and healthy volunteers (each n ≥ 3). Human volunteers were exposed to a single dose of oral losartan (50 mg) and blood was drawn before and at determined intervals after the dose. Serum concentrations are show for **(A)** losartan **(B)** the angiotensin receptor blocking metabolite 3,174, and **(C)** the anti-inflammatory metabolite EXP3179. People with CF off modulators had the lowest serum concentration and all had virtually no EXP3179 detected, which is consistent with 14-week dosing (Table 2).

Pharmacokinetics of oral losartan (50 mg single dose) resulted in serum levels that were low (Figure 2), in agreement with the low airway concentrations of losartan and metabolites found in sheep after 4 days of oral dosing. However, losartan never reached levels needed for meaningful and positive responses of mucociliary clearance parameters *in vitro*, which was 1 µM losartan (Kim et al., 2020; Kim et al., 2022). The predicted losartan levels with 100 mg (maximum FDA approved daily dose), would still fall below 1 µM. Most importantly, EXP3179 levels were basically absent in pwCF off modulators and the highest were found, albeit still at low levels, in pwCF on modulators. Comparing pwCF, subjects off modulators had delayed uptake and lower levels of losartan and its metabolites. This indicates changes in pharmacokinetics likely due to poor absorption related to CF off modulators.

### 3.3 MCC/CC in pwCF study outcomes

Seven participants completed the study, of which five were female. Six were white, non-Hispanic (Table 1). Details on CFTR variants can be found in Supplementary Table S1. The consort study flow diagram is shown in Supplementary Figure S2. Although four participants with F508del mutations were eligible for modulators, these participants were not currently treated with modulators due to previous adverse reactions or personal choice. There were no severe adverse events related to the trial regimen. A summary of adverse events is listed in Supplementary Table S2.

**TABLE 1:** Baseline characteristics of CF participants.

Characteristic (N = 8)	
Age, yrs (mean ± SD)	34.9 ± 9.3
Female, N (%)	5 (63%)
BMI	26.6 ± 4.4
Race/Ethnicity, N (%)	
• White, non-hispanic	6 (75%)
• Black, non-hispanic	1 (12.5%)
• Hispanic/Latino	1 (12.5%)
CFTR mutation, N (%)	
• F508del/F508del	3 (37.5%)
• F508del/other	1 (12.5%)
• Other	4 (50%)
ppFEV1, % (mean ± SD)	75.6 ± 14.6
Pancreatic insufficiency, N (%)	7 (87.5%)
CF-related diabetes (CFRD), N (%)	2 (25%)

BMI, body mass index; ppFEV1 = percent predicted forced expiratory volume in one second; more details on CFTR, variants in Supplementary Table S1.

**TABLE 2:** Serum concentrations of losartan and losartan metabolites.

Losartan and metabolites	Baseline (N = 7)	Week 14 (N = 7)
Losartan, µM	BLQ	0.12 ± 0.14
EXP3179, µM	BLQ	BLQ
EXP3174, µM	BLQ	0.62 ± 0.42

Data are mean ± SD; BLQ: below limit of quantification (<0.003 µM).

#### 3.3.1 Serum concentrations of losartan and metabolites (table 2)

Concentrations of losartan and its metabolites, EXP3174 and EXP3179, were measured from serum samples in pwCF off modulators at baseline and after 14 weeks (n = 6). Losartan, EXP3174, and EXP3179 were not detected in serum at baseline. Losartan was detected at low concentrations at week 14 (0.12 ± 0.14 µM), while EXP3174 was detected at higher concentrations (0.62 ± 0.42 µM), still well below necessary levels seen in the aqueous airway compartment of sheep after inhalation (Figure 1). Most importantly, EXP3179 was not detected in serum samples after 14 weeks.

#### 3.3.2 MCC/CC (figure 3 and table 3)

**FIGURE 3:**
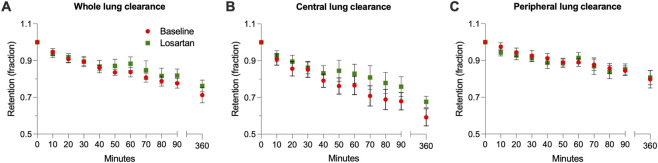
Oral losartan at 100 mg daily for 14 days does not improve mucociliary or cough clearance (MCC/CC). Shown are **(A)** whole lung clearance **(B)** central lung clearance, and **(C)** peripheral lung clearance. None of these measurements differed significantly from baseline after 14-days of daily treatment with oral losartan at 100 mg. All n = 6 pwCF off modulators.

MCC/CC measurements were performed at baseline and after 14 weeks of losartan treatment. However, data from two participants were incomplete because Tc99 m-SC particles failed to deposit in the large airways in one participant and one participant withdrew from the study. Here, we report MCC/CC changes from six participants. No significant difference in whole lung, peripheral, and central MCC (AveClr60) was found between baseline and after 14 weeks of losartan treatment (Figure 3; Table 3). The same was true for cough-assisted clearance (AveClr60-90; Figure 3; Table 3).

**TABLE 3:** Mucociliary clearance/cough clearance (MCC/CC).

MCC/CC	Baseline (N = 6)	Week 14 (N = 6)
Whole lung		
MCC (AveClr60), %	17.2 (CI: 9–25)	15.6 (CI: 0–22)
CC (AveClr60-90), %	5.9 (CI: 0.6–10)	6.8 (CI: −1.8–10)
Central ROI		
MCC (AveClr60), %	26.7 (CI: 8.3–37.2)	20.3 (CI: 1.1–33.5)
CC (AveClr60-90), %	6.8 (CI: 4.3–15.6)	7.6 (CI: 4.1–8.2)
Peripheral ROI		
MCC (AveClr60), %	9.2 (CI: 7.1–21.8)	11.7 (CI: −1.6 – 15.4)
CC (AveClr60-90), %	3.6 (CI: −2.3 – 11.6)	7.1 (CI: −6.9 – 12.5)
C/P ratio	1.6 (CI: 1.1–2.1)	1.4 (CI: 1.1–1.6)

Data are median; CI, 95% confidence interval; MCC, mucociliary clearance; CC, cough clearance; AveClr60 = Median % clearance from 0–60 min; AveClr60-90 = Median % clearance from 60–90 min.

#### 3.3.3 Inflammation markers (n ≥ 6, with one sample being too low amount for all analyses)

**FIGURE 4:**
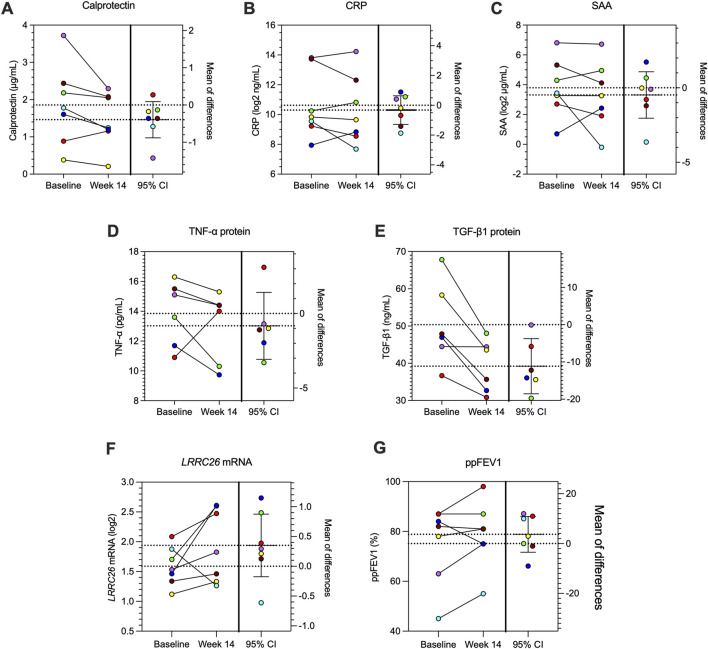
Serum and nasal inflammatory markers and lungs function in study participants. Each participant is color coded throughout this figure. Serum levels of **(A)** calprotectin **(B)** C-reactive protein (CRP), and **(C)** serum amyloid A (SAA). Nasal levels of **(D)** TNF-α and **(E)** TGF-β1 proteins measured in nasal epithelial lining fluid from study participants at baseline and after 14 weeks of losartan treatment. Nasal cell expression of **(F)** *LRRC26* mRNA and **(G)** percent predicted FEV1 (ppFEV1) at baseline and after 14 weeks of losartan treatment. *Statistics:* Before-after plot and 95% CI are shown for each graph. Paired t-test.

Serum calprotectin (Figure 4), serum levels of C-reactive protein (CRP (95% CI, −1.29 to 0.64; p = 0.44) and serum amyloid A (SAA; 95% CI, −2.04 to 1.08; p = 0.48) remained unchanged after 14 weeks of treatment with losartan (Figure 4).

Nasal mucosal samples serve as an accessible surrogate of the lower airways (McDougall et al., 2008; Rebuli et al., 2023). Levels of TNF-α protein measured from nasal epithelial lining fluid decreased in 5 of 6 subjects after 14 weeks of losartan treatment (95% CI, −3.08 to 1.42; p = 0.39) (Figure 4). As losartan is known to have anti-TGF-β1 effects, we also measured TGF-β1 expression in nasal epithelial lining fluid samples. Levels of total TGF-β1 protein were significantly reduced in patients after 14 weeks of losartan (95% CI, −18.55 to −3.75; p = 0.01; Figure 4).

#### 3.3.4 *LRRC26* mRNA expression

Levels of *LRRC26* mRNA expression correlate with BK channel function and TGF-β1 can decrease *LRRC26* expression *in vitro* (Manzanares et al., 2015; Kim et al., 2020). *LRRC26* mRNA expression was measured in nasal epithelial cells by ddPCR and was increased in 6 of 7 participants after 14 weeks of losartan treatment (95% CI, −0.18 to 0.87; p = 0.15; Figure 4).

#### 3.3.5 Pulmonary function

There was no statistically significant change in ppFEV1 in participants after 14 weeks of losartan treatment (Figure 4).

#### 3.3.6 CFQ-R scores

No significant differences between baseline and 14 weeks of losartan were found in any of the 12 domains measured in the CFQ-R (Table 4).

**TABLE 4:** CFQ-R domain scores.

CFQ-R domain	Baseline (N = 7)	Week 14 (N = 7)	Mean change (N = 7)
Physical functioning	72.6 ± 20.5	63.7 ± 27.4	−8.9 (CI: −19.9 to 2.1)
Role perception	82.1 ± 20.1	76.2 ± 17.6	−6.0 (CI: −18.3 to 6.4)
Vitality	54.8 ± 19.2	58.3 ± 21.5	3.6 (CI: −7.2 to 14.3)
Emotion	81.0 ± 20.2	79.1 ± 19.4	−1.9 (CI: −15.6 to 11.8)
Social perception	63.5 ± 13.2	55.6 ± 25.3	−7.9 (CI: 23.6 to 7.7)
Body image	76.2 ± 21.7	76.2 ± 24.4	0.0 (CI: −5.9 to 5.9)
Eating disturbance	85.7 ± 26.2	85.7 ± 15.3	0.0 (CI: −21.4 to 21.4)
Treatment burden	66.7 ± 24.0	61.9 ± 23.0	−4.8 (CI: −14.8 to 5.3)
Health perception	65.1 ± 11.9	68.3 ± 16.3	3.2 (CI: −14.3 to 20.7)
Weight	90.5 ± 25.2	85.7 ± 37.8	−4.7 (CI: −16.4 to 6.9)
Respiratory symptoms	59.5 ± 22.8	59.5 ± 18.4	0.0 (CI: −23.0 to 23.0)
Digestive symptoms	79.4 ± 7.7	82.5 ± 14.1	3.2 (CI: −8.3 to 14.6)

Data are mean ± SD; CI, 95% confidence interval.

## 4 Discussion

Pre-clinical studies demonstrated that losartan and its anti-inflammatory metabolite EXP3179 partially reverse inflammation- and CF-related mucociliary dysfunction by enhancing BK channel activity (Kim et al., 2020; Kim et al., 2022). The losartan concentrations used were 1–10 µM and the resulting EXP3179 concentrations >1 µM. The *in vivo* ovine data suggested that high concentrations reached by nebulizing losartan were excellent at reversing mucociliary dysfunction induced by CFTR inhibition and inflammation by neutrophil elastase (Kim et al., 2020).

Here, we first evaluated the differences in concentrations that can be reached with inhaled vs. oral losartan in sheep. Oral dosing never reached significant airway mucus levels of either losartan or its metabolites, suggesting oral dosing will not be able to improve parameters of mucociliary clearance. The human volunteer pharmacokinetic study showed the same. Serum levels were not reaching concentrations required in the airways to be therapeutic.

We acknowledge that the clinical trial enrolled a small number of participants, which limits statistical power for detecting modest clinical effects. This study was intentionally designed as an exploratory, mechanistic, proof of futility trial focused on determining whether oral losartan could plausibly achieve airway exposure sufficient to affect mucociliary clearance. The consistent absence of therapeutic airway drug concentrations across participants supports the conclusion that oral dosing is unlikely to be effective, even if studied in a larger cohort.

From a pharmacokinetic point of view, pwCF not on modulator therapy tended to have lower levels than those of pwCF on modulators and healthy volunteers. The cause of the observed difference between pwCF with and without modulators is likely multifaceted. Gastrointestinal absorption and hepatic metabolism of drugs is known to be altered in pwCF. However, absorption is expected to improve in response to modulators. Additionally, ivacaftor and its metabolites are known to be a weak inhibitor of CYP3A4 and CYP2C9, the primary enzymes responsible for losartan biotransformation to EXP3174 and EXP3179, and P-glycoprotein, a member of the ATP-binding cassette (ABC) transporter superfamily that can transport losartan (Robertson et al., 2015). Knowledge of the consequences of P-glycoprotein and CYP inhibition on the disposition of losartan and its metabolites may lead to a better understanding of why levels of losartan and metabolites in pwCF on modulators mirror those or are even better than those of healthy volunteers.

Since these preclinical and human volunteer drug level studies were discouraging, the clinical study was designed as an exploratory proof of futility investigation focused on pharmacologic feasibility rather than clinical efficacy. Given the consistently subtherapeutic airway exposure achieved with oral dosing in sheep, larger efficacy trials would not be ethically or scientifically justified. We evaluated if oral losartan could be used to improve MCC/CC and inflammatory markers in pwCF off modulators with a longer duration trial.

In the CF-like sheep model, measured airway concentrations of losartan and its metabolites exceeded levels required to improve mucociliary function in prior *in vitro* studies, validating the relevance of the theoretical deposition estimates. Rapid formation of EXP3179 and EXP3174 within the airway demonstrates active local metabolism. While species specific metabolic differences between sheep and humans may exist, the generation of identical metabolites supports translational relevance of the findings.

As shown in our pharmacokinetic studies, oral losartan and its metabolites did not reach relevant airway and serum concentrations needed in an ovine CF-like model to improve tracheal mucus velocity *in vivo* and parameters of mucociliary function *in vitro* (Kim et al., 2020; Kim et al., 2022). Thus, it was not surprising that our open label clinical trial in 6 pwCF failed to show an increase in any MCC/CC after 14 weeks of treatment with the maximum allowable dose of losartan daily (100 mg). While some improvements in lung function and systemic markers of inflammation were observed in individual participants, it remains unclear if this was related to losartan treatment. In collected nasal fluid, losartan significantly reduced TGF-β1, consistent with reports of its anti-TGF-β1 activity. However, this reduction did not translate to any improvements in the lower airways.

Outside the lungs, trials utilizing losartan for angiotensin-independent purposes have yielded mixed results. An initial trial of losartan in Marfan’s syndrome to slow aortic enlargement found losartan to be beneficial compared to standard therapies (Brooke et al., 2008). However, a larger, multicenter study did not confirm benefit (Lacro et al., 2014). A previous clinical trial using oral losartan as a therapeutic in lung disease also failed to replicate findings in animal models and *in vitro* studies. There, losartan was found to inhibit cigarette smoke-induced TGF-β signaling in a murine model of emphysema and to attenuate the destructive airway enlargement and airway wall thickening in cigarette smoke-exposed mice (Podowski et al., 2012). The follow-up clinical trial to determine the efficacy of oral Losartan Effects on Emphysema Progression (LEEP) and to slow emphysema progression in chronic obstructive pulmonary disease (COPD) showed no efficacy at halting emphysema progression using high-resolution computed tomography over 48 weeks (Wise et al., 2022).

The most likely explanation for the discrepancy between pre-clinical and clinical studies is related to insufficient airway levels achieved by oral dosing of losartan. Dosing of oral losartan is FDA approved up to a maximum of 100 mg daily. We show here that this is insufficient to reach relevant concentrations in serum of pwCF and healthy volunteers. Furthermore, animal studies here also show that oral losartan did not reach levels in the airway that are needed to have effects on parameters of mucociliary clearance. This is also true for the losartan metabolites, especially for the anti-inflammatory metabolite EXP3179.

Future studies powered for clinical efficacy would only be justified if alternative delivery strategies capable of achieving adequate airway exposure are employed. This would likely involve altering delivery route to ensure that the lung is exposed to adequate concentrations of losartan and its relevant metabolites (especially the anti-inflammatory EXP3179). Alternatively, exploring clinical trial designs utilizing target-concentration interventions could be another strategy to ensure adequate systemic exposure, pushing doses above the FDA approved maximum. However, our data indicate that the latter would be difficult to achieve without significant systemic side effects.

## Data Availability

The raw data supporting the conclusions of this article will be made available by the authors, without undue reservation.
