# Supplementary material for: Oral losartan yields subtherapeutic airway exposure compared to nebulized delivery and fails to improve mucociliary clearance in cystic fibrosis
Source: Front Pharmacol. 2026 May 4;17:1807581. doi: 10.3389/fphar.2026.1807581 (PMC13180560; doi:10.3389/fphar.2026.1807581)
Supplement: Supplementary file 1 [file DataSheet1.pdf]

Supplementary Material for

Oral losartan yields subtherapeutic airway exposure compared to nebulized delivery and fails to improve mucociliary clearance in cystic fibrosis

Charles D. Bengtson[1,#], Andreas Schmid[1,#], Michael D. Kim[1,#], John S. Dennis[1], J. Steven Leeder[2], Kirby L. Zeman[3], William Bennett[3], Juan Sabater[4], Nathalie Baumlin[5], Matthias Salathe[5,*]

[1]Department of Internal Medicine, Division of Pulmonary Critical Care and Sleep Medicine, University of Kansas Medical Center, Kansas City, KS 66160, USA

[2]Department of Pediatrics and Children's Mercy Research Institute, Children's Mercy Hospital, Kansas City, MO 64108, USA

[3]Department of Medicine, University of North Carolina at Chapel Hill, Chapel Hill, NC 27599, USA

[4]Departemnt of Research, Mount Sinai Medical Center, Miami Beach, FL 33140, USA

[5]Department of Internal Medicine, University of Kansas Medical Center, Kansas City, KS 66160, USA

# Contributed equally

1 Supplementary Materials and Data

1.1 Inclusion and exclusion criteria.

Inclusion criteria

- Cystic fibrosis (CF) patient ≥18 years of age with any known mutation combination not on CFTR augmentation therapy (females and males)
- Severity of the disease: Suitable patients will have mild to moderate lung disease, as defined by:
  - Pulmonary function: ppFEV1 ≥40% at screening
  - Hemoglobin oxygen saturation of >92% on room air by pulse oximetry at the screening visit
  - Produces sputum regularly (daily basis, at minimum)
- $ppFEV_1$ ≥ 40% of predicted at screening visit
- Informed consent – patient agrees to participation in the study by signing and dating the informed consent form after the nature of the study has been fully explained and all questions have been satisfactorily answered.
- Negative COVID-19 test within 72 hours prior to MCC/CC testing

Exclusion criteria

- When enrolling female patients:
  - Not willing to adhere to strict birth control (combination of two methods)
  - If female, patient must be non-pregnant and non-lactating, and those of childbearing potential must be using an acceptable method of birth control (i.e., an intrauterine contraceptive device with a failure rate of <1%, hormonal contraceptives or a barrier method). If a female patient is abstinent, she must agree to use one of the acceptable methods if she becomes sexually active.
- Unstable lung disease: As defined by a change in medical regimen during the preceding 2 weeks or a $ppFEV_1$ ≥15% below average of three months prior to enrollment
- Received an investigational drug or therapy during the preceding 30 days
- Active or former smokers with less than 1 year since quitting, or >10 pack-year smoking history
- Unable to adequately complete study measures, including spirometry
- Intolerance to ARBs and/or treatment with ACE inhibitor
- Regular use of NSAIDs or potassium supplementation, treatment with aliskiren
- Oral corticosteroid use within 6 weeks
- Exacerbation requiring treatment within 6 weeks
- Treatment of non-tuberculous mycobacterial lung infections (initiation within the past three months, declining lung function on treatment or use of rifampin in treatment regimen)
- Significant hypoxemia (oxygen saturation <92% on room air and rest or use of continuous oxygen treatment), chronic respiratory failure by history (pCO_2 > 45 mmHg), clinical evidence of cor pulmonale
- Untreated arterial hypertension (systolic >140 mm Hg, diastolic > 90 mmHg)
- Blood pressure less than 90 mm Hg systolic while standing
- Cardiac, renal (creatinine 1.5 times normal limit), hepatic (LFTs > 3x normal upper limit), neurological, psychiatric, endocrine or neoplastic diseases that are judged to interfere with participation in study. In case serum alkaline phosphatase (ALP) is raised 3X normal upper limit, then ALP isoenzymes would be done to confirm the source (liver or bone). Participants will be excluded if the ALP isoenzymes from liver are raised 3X the normal upper limit.

- Known renal artery stenosis
- Concomitant airway disorders other than CF, such as ABPA
- Subjects with prior thoracic surgery
- Other severe acute or chronic medical or psychiatric condition or laboratory abnormality that may increase the risk associated with trial participation or may interfere with the interpretation of trial results and, in the judgment of the PI, would make the subject inappropriate for enrollment.
- Patients using intermittent inhaled or oral antibiotics will be allowed to participate in this trial. Patients on chronic, cycling antibiotics will be required to have completed at least 2 full cycles of the prescribed antibiotic prior to enrollment and should be studied during the same phase of treatment (on or off) during each study period.
- Radiation exposure within the past year that would cause them to exceed Federal Regulations by participating in this study.

2 Supplementary Figures and Tables

2.1 Supplementary Figures

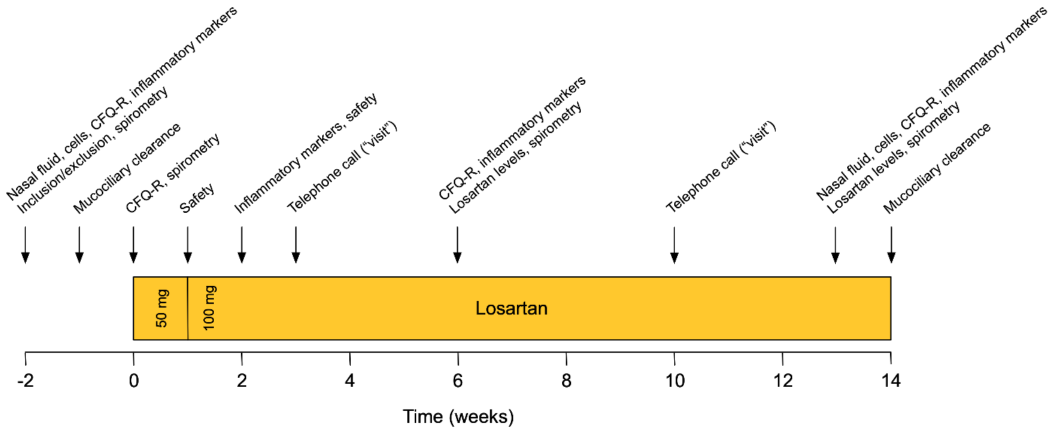

Supplementary Figure S1. Clinical trial design.

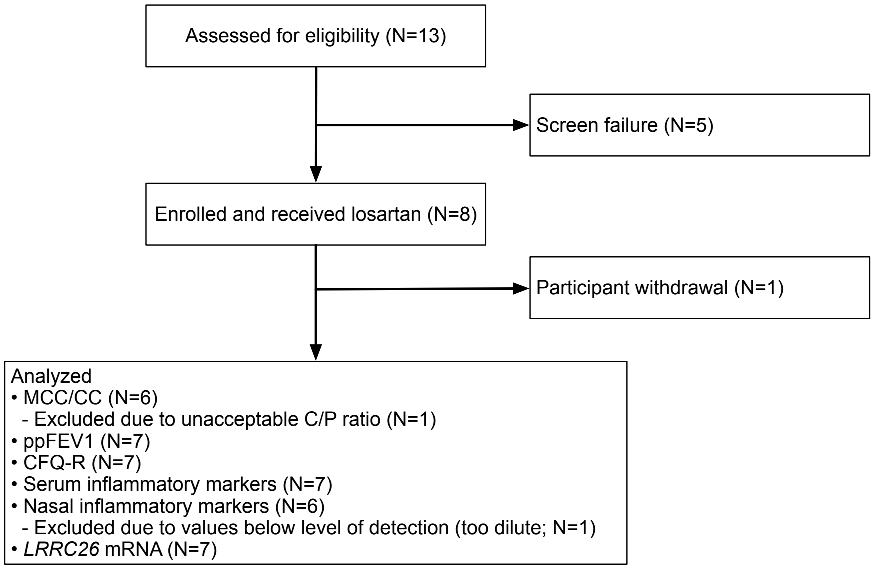

Supplementary Figure S2. Consort diagram of enrollment and analysis of the losartan trial.

2.2 Tables

Supplemental Table S1. CFTR variants of the 8 participants

CFTR variant	CFTR variant
1898+1G->A	V520F
1898+1G->A	V520F
935delA	935delA
L467P	S434X
F508del	2184insA
F508del	F508del
F508del	F508del
F508del	F508del

Supplemental Table S2. Summary of adverse events.

**Event**	**No. of Participants (N=8)**
Any adverse event	8 (100%)
Adverse event related to trial regimen	3 (37.5%)
Maximum severity of adverse event	
Mild	2 (25%)
Moderate	6 (75%)
Severe	0 (0%)
Adverse event leading to interruption of trial regimen	1 (12.5%)
Adverse event leading to discontinuation of trial regimen	0 (0%)
Most common adverse event	
Pulmonary exacerbation	5 (62.5%)
Elevated creatinine	2 (25%)
Elevated liver enzymes	2 (25%)
Elevated creatinine kinase	1 (12.5%)
Muscle pain	1 (12.5%)
Tinea pedis	1 (12.5%)
Dysuria	1 (12.5%)
Fatigue	1 (12.5%)
Constipation	1 (12.5%)
Abdominal pain	1 (12.5%)
Vomiting	1 (12.5%)
Dizziness	1 (12.5%)
Recurrent jugular vein thrombosis	1 (12.5%)
Hypoglycemia	1 (12.5%)
Anemia	1 (12.5%)
